# In Support of *EHP*’s Proposal to Adopt the ARRIVE Guidelines

**DOI:** 10.1289/ehp.1307775

**Published:** 2013-12-01

**Authors:** Hanna M. Vesterinen, Paula I. Johnson, Erica Koustas, Juleen Lam, Patrice Sutton, Tracey J. Woodruff

**Affiliations:** 1 Program on Reproductive Health and the Environment, University of California, San Francisco, Oakland, California, USA; 2 Oak Ridge Institute for Science and Education, Washington, DC, USA; 3 Department of Health Policy and Management, Johns Hopkins Bloomberg School of Public Health, Baltimore, Maryland, USA, E-mail: VesterinenH@obgyn.ucsf.edu

We strongly support *EHP*’s proposal encouraging authors who perform animal studies to adhere to the ARRIVE guidelines (http://www.nc3rs.org.uk/ARRIVE/) (Tilson and Schroeder 2013), a 20-item checklist for the reporting of key elements necessary to describe a study comprehensively and transparently (Kilkenny et al. 2010). In the clinical sphere, systematic review methods have been at the forefront of evidence-based research for the past 20 years and have demonstrated that poor reporting, particularly in domains associated with increased risk of bias, can have a significant impact on the results of a study (Juni et al. 2001). By supporting the ARRIVE guidelines, *EHP* recognizes the importance of reporting—and ultimately implementing—methodological approaches that can influence study quality. The ARRIVE guidelines have already been adopted by many journals, including several high-impact publications from the Nature, BioMed Central, and PLoS publishing groups. *EHP* will join these other prestigious journals in leading the way in recognizing the importance of reporting methodological elements in the toxicological sciences and ultimately strengthening the scientific approach.

Although we endorse the use of an approach such as the ARRIVE guidelines, the ARRIVE guidelines do not currently include all important reporting elements in a clearly defined manner. In addition, because the ARRIVE guidelines were developed in the clinical sphere, some elements that are pertinent to toxicological research are not included. We encourage *EHP* to simultaneously address those issues, including the following:

Authors should adequately report sample sizes per group (Landis et al. 2012) and include explicit details of any losses to follow-up. We urge *EHP* to encourage authors to perform and report details of an *a priori* sample size calculation.
Authors should describe any animals with “peculiarities”; this criterion is described in the “Gold Standard Publication Checklist” by Hooijmans et al. (2010) but is not necessarily covered by the “adverse events” criteria in the ARRIVE guidelines.
Authors should report where and when the study was performed to aid in assessing whether the cohort of animals is unique from other published studies.
Whenever possible, authors should include in the study doses that are environmentally relevant (to humans) and a measured concentration in the animal for comparison/integration with human biomarkers.

Authors should also report all of their funding sources and include a statement regarding potential conflicts of interest, including when none exist. “Conflict of interest” risk-of-bias domain has been proposed—but not yet adopted—by the Cochrane Collaboration and GRADE (Grading of Recommendations Assessment, Development and Evaluation) as an important risk of bias. This is based on empirical data from studies of the health effects of tobacco (Barnes and Bero 1997, 1998), the safety and efficacy of pharmaceuticals (Bero et al. 2007; Lexchin et al. 2003; Lundh et al. 2012), and medical procedures (Popelut et al. 2010; Shah et al. 2005) that have shown that source of funding influences the study outcome.

A criticism of the ARRIVE guidelines is that they are not topic specific. However, we believe that risk-of-bias domains used in human experimental studies that have an empirical basis—including sequence generation, allocation concealment, blinding, incomplete outcome data, and selective reporting—are directly relevant to toxicological studies. In the clinical sphere, these five criteria address nearly all issues that bear on the quality of human experimental evidence (Balshem et al. 2011). Further, these elements have been shown in the preclinical animal literature to influence study outcomes (Landis et al. 2012; Vesterinen et al. 2010). Therefore, in the absence of evidence to the contrary, the field of toxicology should proceed based on such comparability. At the same time, it would be of enormous benefit to the field of toxicology research for *EHP* to adopt the ARRIVE guidelines and to simultaneously encourage empirically based research to assess which criteria are most critical to the results of various types of toxicological studies that provide the evidence for decision making in environmental health.

Finally, for the *EHP* editors to highlight their commitment to improving the quality of the research published in *EHP*, they could commission or encourage research that assesses the current standard or quality of their publications and then repeat the exercise in a few years to see if the guidelines have resulted in improvements to both reporting and quality of toxicological studies [for example, see Dirnagl and Lauritzen (2011)].
